# What is the added value of CT-angiography in patients with transient ischemic attack?

**DOI:** 10.1186/s12883-021-02523-y

**Published:** 2022-01-03

**Authors:** Ilko L. Maier, Gerrit U. Herpertz, Mathias Bähr, Marios-Nikos Psychogios, Jan Liman

**Affiliations:** 1 Department of Neurology, University Medicine Göttingen, Robert-Koch-Str. 40, 37075 Göttingen, Germany; 2 Department of Anesthesiology, Klinikum Bremerhaven-Reinkenheide, Bremerhaven, Germany; 3 Department of diagnostic and interventional Neuroradiology, University Clinic Basel, Basel, Switzerland

## Abstract

**Background:**

Transient ischemic attack (TIA) is an important predictor for a pending stroke. Guidelines recommend a workup for TIA-patients similar to that of stroke patients, including an assessment of the extra- and intracranial arteries for vascular pathologies with direct therapeutic implications via computed tomography angiography (CTA). Aim of our study was a systematic analysis of TIA-patients receiving early CTA-imaging and to evaluate the predictive value of TIA-scores and clinical characteristics for ipsilateral vascular pathologies and the need of an invasive treatment.

**Methods:**

We analysed clinical and imaging data from TIA patients being admitted to a tertiary university hospital between September 2015 and March 2018. Following subgroups were identified: 1) no- or low-grade vascular pathology 2) ipsilateral high-risk vascular pathology and 3) high-risk findings that needed invasive, surgical or interventional treatment. We investigated established TIA-scores (ABCD_2_-, the ABCD_3_- and the SPI-II score) and various clinical characteristics as predictive factors for ipsilateral vascular pathologies and the need for invasive treatment.

**Results:**

Of 812 patients, 531 (65.4%) underwent initial CTA in the emergency department. In 121 (22.8%) patients, ipsilateral vascular pathologies were identified, of which 36 (6.7%) needed invasive treatment. The ABCD_2_-, ABCD_3_- and SPI-II-scores were not predictive for ipsilateral vascular pathologies or the need for invasive treatment. We identified male sex (OR 1.579, 95%CI 1.049–2.377, *p* = 0.029), a short duration of symptoms (OR 0.692, 95% CI 0.542–0.884, *p* = 0.003), arterial hypertension (OR 1.718, 95%CI 0.951–3.104, *p* = 0.073) and coronary heart disease (OR 1.916, 95%CI 1.184–3.101, *p* = 0.008) as predictors for ipsilateral vascular pathologies. As predictors for the need of invasive treatment, a short duration of symptoms (OR 0.565, 95%CI 0.378–0.846, *p* = 0.006), arterial hypertension (OR 2.612, 95%OR 0.895–7.621, *p* = 0.079) and hyperlipidaemia (OR 5.681, 95%CI 0.766–42.117, *p* = 0.089) as well as the absence of atrial fibrillation (OR 0.274, OR 0.082–0.917, *p* = 0.036) were identified.

**Conclusion:**

More than every fifth TIA-patient had relevant vascular findings revealed by acute CTA. TIA-scores were not predictive for these findings. Patients with a short duration of symptoms and a vascular risk profile including coronary heart disease, arterial hypertension and hyperlipidaemia most likely might benefit from early CTA to streamline further diagnostics and therapy.

**Supplementary Information:**

The online version contains supplementary material available at 10.1186/s12883-021-02523-y.

## Background

TIA is a well-known predictor for a manifest ischemic stroke with an incidence over 10% within 3 months after the index TIA. Nearly half of ischemic strokes occur during the first 2 days after the index TIA [1]. Scores like the ABCD_2_-Score [2], the ABCD_3_-Score [3] and the Stroke Prognosis Instrument-II (SPI-II) [4] have been developed to quantify the risk of a pending stroke after TIA. To reduce the risk for a pending ischemic stroke following a TIA, it is important to perform a full diagnostic workup to identify common aetiologic causes like ipsilateral vascular stenosis or occlusion. With a consequent workup and early treatment in specialised stroke centers, the risk for recurring strokes can be reduced below 3.5% after 90 days [5, 6].

Patients with TIA, owing to intrinsic thrombolytic mechanisms and cerebral collateral status, can present with transient ischemic symptoms despite severe proximal stenosis, dissection of brain supplying vessels, acute occlusion of pre-existing stenosis or as a result of an acute embolic occlusion of large cerebral vessels [7]. In this respect, current guidelines recommend managing TIA-patients the same way as patients with a manifest stroke in the emergency department, which include that a computed tomography angiography (CTA) of the brain supplying arteries should be considered [8–10]. Macroangiopathic causes like arterial stenosis or occlusion account for around 23% of all TIA-cases, which reliably can be detected using CTA [11, 12]. Hereby, vascular findings in the CTA allow preventive measures and may have a direct impact on the acute treatment of the TIA-patient e. g. for a hemodynamically relevant dissection, intravascular thrombi, or an acute large vessel occlusion. Only the targeted diagnosis of one of those pathologies justifies the performance of a CTA, whose possible side effects include the risk of nephrotoxicity (in 2% of cases) and the risk of an allergic reaction to contrast agent (in 0.01% of cases) as well as additional radiation exposure which is two times higher than that of a native CT-scan [13–15].

The aim of our study was to perform a systematic evaluation of the role of CTA imaging in the acute setting of TIA, to evaluate which subgroups of TIA-patients benefit from initial imaging with CTA and to investigate the predictive value of TIA-scores regarding ipsilateral vascular pathologies.

## Methods

### Patient population and study design

**Fig. 1:**
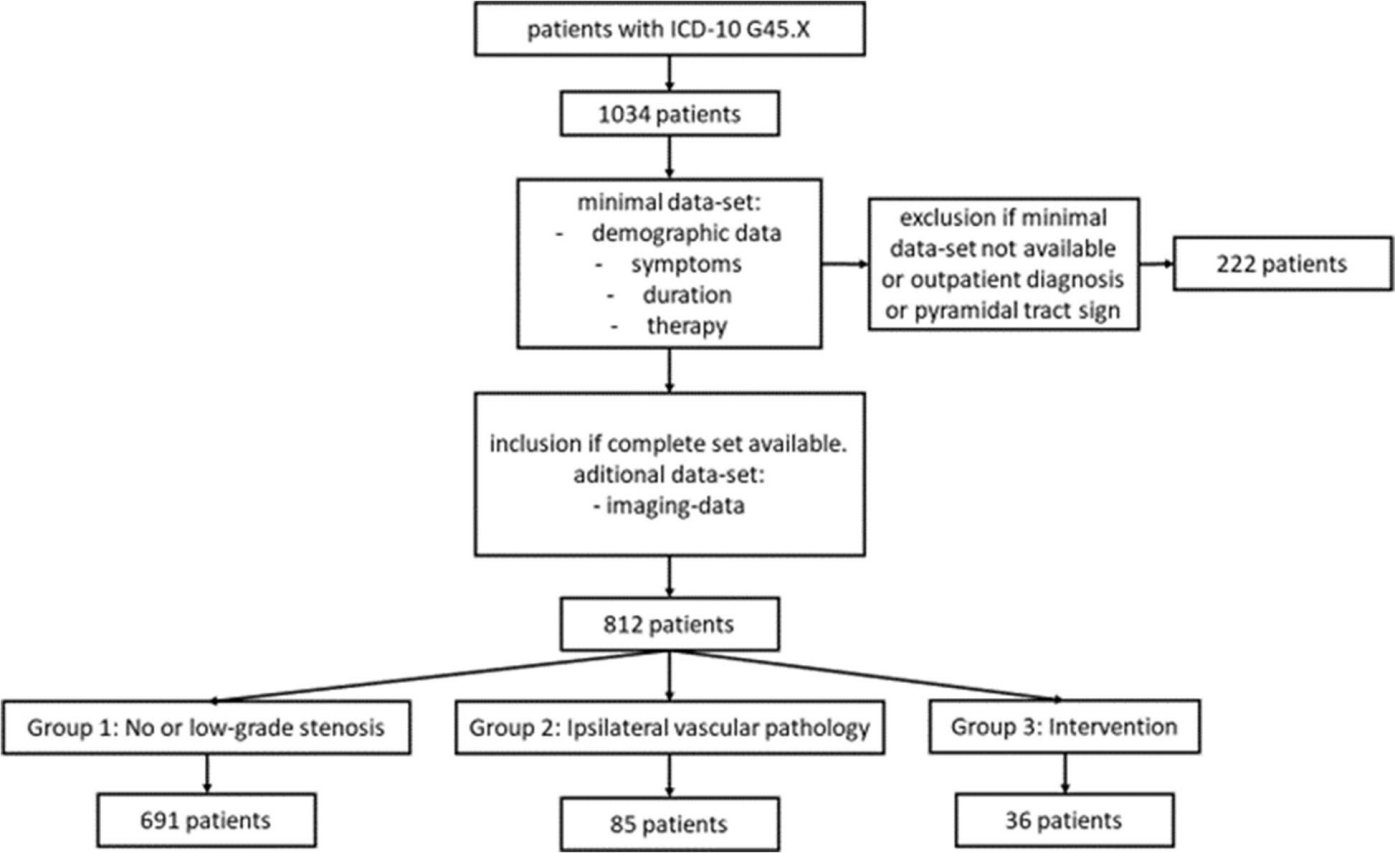
Inclusion flow chart

In this retrospective study, clinical and imaging data was collected from patients with the admission diagnosis TIA, who were treated in a tertiary university hospital between September 2015 and March 2018. From all identified patients with TIA as their admission diagnosis, a total number of 222 patients were excluded for the following reasons (see Fig. 1): 74 patients had insufficient data (minimal dataset not available), 50 patients had a change of diagnosis during in hospital stay (e.g. TIA-mimics like intoxication or hypoglycaemia), 55 patients were follow-up cases in our vascular outpatient clinic and not admitted via the emergency department, 22 patients refused treatment during in-patient stay and did not receive full workup and 21 patients presented with pyramidal tract signs (like Babiski’s-, Gordon’s- or Oppenheim’s sign), which are more likely to indicate an ischemic stroke rather than a TIA.

The remaining 812 TIA-patients were categorised into three subgroups: 1) no or low-grade vascular pathology, 2) high-grade, ipsilateral vascular pathology (highly likely to have caused the TIA) and 3) patients with the need for invasive treatment (e.g. surgically or interventionally). A low-grade vascular pathology was considered, if it occluded less than 50% of the vessel, according to the North American Symptomatic Carotid Endarterectomy Trial (NASCET) [16]. Vascular pathologies with a stenosis over 50% (NASCET) were considered as high-grade. Patients were categorized in the second group (ipsilateral vascular pathology), if high-risk pathologies like high-grade stenosis (> 50% NASCET), vascular dissection, complete occlusions or intraarterial thrombi ipsilateral to the affected territory were diagnosed. All vascular pathologies were either diagnosed by CTA in the emergency department in combination with consecutive neurovascular ultrasound or by neurovascular ultrasound alone. Neurovascular ultrasound of all accessible cerebral vessels was performed by experienced neurologists and CTA were reviewed by experienced neuroradiologists. Patients receiving invasive treatment like intravenous thrombolysis (IVT), mechanical thrombectomy (MT), surgical (carotid endarterectomy (CEA)) or interventional treatment (e.g. carotid artery stenting (CAS)) were assigned to group 3.

Data was obtained from the emergency medical service documentation, the clinics digital patient documentation system ixserv (ixmid Software Technologie GmbH) and the intensive care information system IntelliSpace Critical Care and Anaesthesia (Philips). The collected data included baseline characteristics like age and sex, initial assessment, kind of symptoms, symptom duration and National Institute of Health Stroke Scale (NIHSS), lab results, patient’s medical history and pre-medication. The results of stroke-imaging and neurovascular ultrasound were included. Finally, we documented if the patients received IVT, MT, CEA, CAS or carotid percutaneous transluminal angioplasty without stenting (PTA only). From all available data we calculated every patients ABCD_2_-score, ABCD_3_-score and Stroke Prognosis Instrument II [2–4]. The ABCD_2_-score includes the items age, blood pressure, neurologic symptoms, duration of symptoms and diabetes mellitus. The ABCD_3_-score adds the item TIA within the last 7 days. The SPI-II focusses more on pre-existing conditions like heart failure, coronary heart disease, history of stroke and diabetes. It is completed by the items age, blood pressure and duration of symptoms longer than 24 h.

### Statistical analysis

Statistical analysis was performed using SPSS 21 (IBM SPSS Statistics, Armonk, NY, USA). Baseline characteristics were described using frequencies, means and median with standard deviation and interquartile range, as applicable. Predefined groups (group 1: no or low-grade vascular pathology, group 2: high-grade, ipsilateral vascular pathology and group 3: need for invasive treatment) were compared using T-tests, Wilcoxon-Mann-Whitney tests and χ^2^ test, as appropriate. Predictors for ipsilateral vascular pathologies were included in uni- and multivariate logistic regression models. To create predictive models for the endpoints ‘ipsilateral vascular pathology’ and ‘need for invasive treatment’ we selected predictors, if 1) they were clinically plausible, 2) they were known at baseline (e.g. from history-taking or clinical examination and 3) they did not cause multicollinearity within the model. For the multivariate, binary logistic regression analysis, the backward selection function with a removal cut-off from the model of *p* < 0.1 has been used to identify the factors with the highest predictive value. To evaluate the predictive value of the multivariate logistic regression models and the TIA scores, Area Under the Receiver Operator Curves (AUROC) and confidence intervals (CIs) were calculated.

## Results

In total, 1034 patients with the admission diagnosis ‘TIA’ were screened, of which 812 were ultimately included in the study. Of these 812 patients, 531 (65.4%) received immediate CTA in the emergency department. Baseline characteristics are given in Table 1 and the symptoms present on first examination are described in supplementary Table 1.

**Table 1:** Baseline characteristics of TIA-patients with- and without ipsilateral vascular pathology and the need for invasive treatment (*n* = 812)

	No ipsilateral vascular pathology (*n* = 691)	Ipsilateral vascular pathology (*n* = 121)	*P*-value	No invasive treatment (*n* = 776)	Invasive treatment (*n* = 36)	*P*-value
Demographic data						
Age, years ± SD	71 ± 14	71 ± 13	0.760	71 ± 14	68 ± 11	0.165
Sex (male, %)	365 (52.8)	78 (64.5)	0.018	420 (54.1)	23 (63.9)	0.305
Clinical presentation						
Systolic blood pressure (median mmHg, IQR)	150 (135–168)	148 (131–168)	0.789	150 (132–169)	149 (136–154)	0.495
Diastolic blood pressure (median mmHg, IQR)	80 (70–90)	80 (66–90)	0.078	80 (70–90)	80 (65–90)	0.416
NIHSS (median, IQR)	0 (0–1)	0 (0–1)	0.270	0 (0–1)	0 (0–0.75)	0.097
Duration of symptoms			0.020			0.012
< 10 min	107 (15.5)	31 (25.6)		126 (16.2)	12 (33.3)	
< 60 min	163 (23.6)	28 (23.1)		181 (23.3)	10 (27.8)	
> 60 min	421 (60.9)	62 (51.2)		469 (60.4)	14 (38.9)	
Co-morbidities						
Arterial hypertension (n, %)	551 (79.7)	106 (87.6)	0.042	625 (80.5)	32 (88.9)	0.213
Hyperlipidaemia (n, %)	590 (85.4)	108 (89.3)	0.258	663 (85.4)	35 (97.2)	0.047
Atrial fibrillation (n, %)	148 (21.4)	29 (24.0)	0.531	174 (22.4)	3 (8.3)	0.045
Diabetes mellitus (n, %)	166 (24)	29 (24)	0.989	187 (24.1)	8 (22.2)	0.797
Obesity (n, %)	119 (17.2)	26 (21.5)	0.258	134 (17.3)	11 (30.6)	0.042
Coronary heart disease (n, %)	97 (14)	31 (25.6)	0.001	121 (15.6)	7 (19.4)	0.535
Heart failure (n, %)	91 (13.2)	18 (14.9)	0.611	104 (13.4)	5 (13.9)	0.933
History of myocardial infarction (n, %)	48 (6.9)	20 (16.5)	< 0.001	6.4 (8.2)	4 (11.1)	0.544
Aortic valve stenosis (n, %)	38 (5.5)	8 (6.6)	0.625	44 (5.7)	2 (5.6)	0.977
Chronic kidney disease (n, %)	104 (15.1)	22 (18.2)	0.380	120 (15.5)	6 (16.7)	0.846
PFO (n, %)	119 (17.2)	15 (12.4)	0.187	130 (16.8)	4 (11.1)	0.373
PFO + atrial aneurysm (n, %)	23 (3.3)	0 (0)	0.042	23 (3)	0 (0)	0.295
Pulmonal embolism (n, %)	8 (1.2)	2 (1.7)	0.649	23 (3)	0 (0)	0.295
History of cancer (n, %)	74 (10.7)	13 (10.7)	0.991	84 (10.8)	3 (8.3)	0.637
History of ischemic stroke (n, %)	137 (19.8)	33 (27.3)	0.063	162 (20.9)	8 (22.2)	0.846
TIA within the last seven days (n, %)	85 (12.3)	20 (16.5)	0.201	96 (12.4)	9 (25.0)	0.027
Medication						
Antiplatelet medication (n, %)	250 (36.2)	70 (57.9)	< 0.001	299 (38.5)	21 (58.3)	0.017
Dual antiplatelet medication (n, %)	15 (2.2)	10 (8.3)	< 0.001	24 (3.1)	1 (2.8)	0.915
NOAK (n, %)	50 (7.2)	8 (6.6)	0.806	56 (7.2)	2 (5.6)	0.705
Marcumar (n %)	66 (9.6)	14 (11.6)	0.429	78 (10.1)	2 (5.6)	0.376
Antihypertensive medication (n, %)	508 (73.5)	100 (82.6)	0.033	578 (74.5)	30 (83.3)	0.231
Antihyperlipidemic medication (n, %)	246 (35.6)	69 (57)	< 0.001	295 (38)	20 (55.6)	0.035

*SD* Standard deviation, *IQR* Interquartile range, *NIHSS* National Institute of Health Stroke Scale, *PFO* Persistent foramen ovale, *TIA* Transient ischemic attack, *NOAK* New oral anticoagulant

TIA-patients with ipsilateral vascular pathologies were more likely to be male (64.5% vs. 52.8%, *p* = 0.018), to have a history of arterial hypertension (87.6 vs. 79.7, *p* = 0.042), coronary heart disease (25.6% vs. 14%, *p* = 0.001), myocardial infarction (16.5% vs. 6.9%, *p* < 0.001) and ischemic stroke (27.3% vs. 19.8%, *p* = 0.063). Patients with the need for invasive treatment were more likely to have hyperlipidaemia (97.2% vs. 85.4%, *p* = 0.047), to be obese (30.6% vs. 17.3%, *p* = 0.042) and to have had a TIA within 7 days prior to the index TIA (25% vs. 12.4%, *p* = 0.027) as well as less likely to have atrial fibrillation (8.3% vs 22.4%, *p* = 0.045). Both patients with ipsilateral vascular pathologies and patients with the need for invasive treatment had a lower duration of TIA-symptoms (with highest differences in the group of patients with a symptom duration of < 10 min) and were more likely to be on antiplatelet- and lipid lowering medication. There were no significant differences concerning presenting symptoms between the groups with- and without ipsilateral vascular pathologies, while patients with the need for invasive treatment were more likely to present with hemiparesis (52.8% vs. 34.7, *p* = 0.026) and amaurosis fugax (19.4% vs. 9.5%, *p* = 0.052) and were less likely to present with dysarthria (8.3% vs. 22.9%, *p* = 0.040).

Using the imaging data of the 531 patients receiving a CTA in the emergency department, we found that 121 (22.8%) patients showed a high-grade ipsilateral arterial pathology: 91 (17.1%) were found with high-grade, ipsilateral arterial stenosis, 20 (3.8%) patients with a haemodynamic relevant, ipsilateral arterial occlusion, 6 (1.1%) patients with an arterial dissection and 2 (0.4%) patients with instable intraarterial thrombi. Of the 121 patients with high-grade ipsilateral arterial pathology, 36 (6.8%) received invasive treatment: 19 (3.6%) patients received CEA, 9 (1.7%) received CAS, 4 (0,7%) patients received PTA without stenting, 3 (0.6%) patients received an IVT and one (0.2%) patient received subclavian-carotid bypass surgery. None of the patients received an MT based on the acute CTA. More than half of the interventions were performed within 1 week after the CTA and all invasive treatments were performed during the in-patient stay after the index TIA within a maximum period of 2 weeks. There was no immediate intervention after the CTA, not taking IVTs into consideration. In contrast, of all 281 patients without early CTA, 3 (1.1%) patients were diagnosed with high-grade ipsilateral vascular pathology (stenosis) via neurovascular ultrasound during in-hospital stay; one (0.4%) patient underwent CEA.

Using the ABCD_2_-, ABCD_3_- and the SPI-II-Score, we found no significant difference between patients with- and without ipsilateral vascular pathologies or the need for invasive treatment (Table 2). These TIA-scores sowed no predictive value for both endpoints in the univariate- and multivariate logistic regression analysis corrected for possible confounders (supplementary Table 2).

As shown in Table 3, multivariate logistic regression models were created to identify predictive factors for ipsilateral vascular pathologies and the need for invasive treatment using demographic and anamnestic data alone (see Tables 1 and 2)- and in combination with presenting symptoms (see supplementary Table 1). For ipsilateral vascular pathology, male sex (OR 1.579, 95%CI 1.049–2.377, *p* = 0.029), a short duration of symptoms (OR 0.692, 95%CI 0.542–0.884, *p* = 0.003), arterial hypertension (OR 1.718, 95%CI 0.951–3.104, *p* = 0.073) and coronary heart disease (OR 1.916, 95%CI 1.184–3.101, *p* = 0.008) were identified as predictors. Including also presenting symptoms in the analysis, the presence of vertigo remained as an additional significant predictor (OR 1.587, 95%CI 1.001–2.516, *p* = 0.049). Predictive factors for the need for an invasive treatment were a short duration of symptoms (OR 0.565, 95%CI 0.378–0.846, *p* = 0.006), arterial hypertension (OR 2.612, 95%CI 0.895–7.621, *p* = 0.079), hyperlipidaemia (OR 5.681, 95%CI 0.766–42.117, *p* = 0.089) and the absence of atrial fibrillation (OR 0.274, 95%CI 0.082–0.917, *p* = 0.036). Including presenting symptoms, hemiparesis as one presenting symptom (OR 2.659, 95%CI, 1.325–5.337, *p* = 0.006) and the absence of dysarthria (OR 0.321, 95%CI 0.096–1.078, *p* = 0.066) remained in the predictive model.

**Table 2:** Transient ischemic attack scores in patients with- and without ipsilateral vascular pathology and the need for invasive treatment

TIA Scores	No ipsilateral vascular pathology (*n* = 691)	Ipsilateral vascular pathology (*n* = 121)	*P*-value	No invasive treatment (*n* = 776)	Invasive treatment (n = 36)	*P*-Value
ABCD_2_-Score (median, IQR)	4 (3–5)	4 (3–5)	0.478	4 (3–5)	4 (3–5)	0.941
ABCD_3_-Score (median, IQR)	4 (3–6)	5 (3–6)	0.757	4 (3–6)	5 (3.25–6)	0.287
SPI-II (median, IQR)	3 (0–5)	3 (1–6)	0.142	3 (1–5)	2.5 (0.25–5)	0.703

*TIA* Transient ischemic attack, *IQR* Interquartile range, *SPI-II* Stroke prognosis instrument

Predictive values for multivariate models in Table 3 for the endpoint ‘ipsilateral vascular pathology’ (model excluding presenting symptoms: AUROC, 0.63; 95%CI, 0.577–0.686, *p* < 0.001; model including presenting symptoms: AUROC, 0.646; 95%CI 0.592–0.699, *p* < 0.001) and the endpoint ‘need for invasive treatment’ (model excluding presenting symptoms: AUROC, 0.722; 95%CI 0.633–0.811, *p* < 0.001; model including presenting symptoms: AUROC, 0.765, 95%CI, 0.683–0.846) were moderate. All TIA scores were not predictive for the endpoints ‘ipsilateral vascular pathology’ and ‘need for invasive treatment’ alone (AUROC < 0.6, *p* = n.s.) and performance was not improved after forcing the TIA scores in the clinical prediction models (data not shown).

**Table 3:** Multivariate logistic regression models including predictors for ipsilateral vascular pathologies and the need for invasive treatment in patients with transient ischemic attack

	Regression coefficient	OR	95%CI	*P*-value
Ipsilateral vascular pathology (model excluding presenting symptoms)				
Sex, male	0.457	1.579	1.049–2.377	0.029
Duration of Symptoms	−0.368	0.692	0.542–0.884	0.003
Arterial Hypertension	0.541	1.718	0.951–3.104	0.073
Coronary heart disease	0.650	1.916	1.184–3.101	0.008
Intercept	−1.723	0.179		< 0.001
Ipsilateral vascular pathology (model including presenting symptoms)				
Sex, male	0.433	1.542	1.023–2.326	0.039
Duration of Symptoms	−0.402	0.669	0.522–0.857	0.001
Arterial Hypertension	0.561	1.753	0.968–3.173	0.064
Coronary heart disease	0.662	1.939	1.196–3.145	0.007
Vertigo	0.462	1.587	1.001–2.516	0.049
Intercept	−1.751	0.174		< 0.001
Need for invasive treatment (model excluding presenting symptoms)				
Duration of symptoms	−0.570	0.565	0.378–0.846	0.006
Arterial Hypertension	0.960	2.612	0.895–7.621	0.079
Hyperlipidaemia	1.737	5.681	0.766–42.117	0.089
Atrial fibrillation	−1.293	0.274	0.082–0.917	0.036
Intercept	−4.023	0.018		0.001
Need for invasive treatment (model including presenting symptoms)				
Duration of symptoms	−0.600	0.549	0.363–0.892	0.004
Arterial Hypertension	0.971	2.639	0.896–7.774	0.078
Hyperlipidaemia	1.761	5.821	0.781–43.363	0.086
Atrial fibrillation	−1.323	0.266	0.079–0.901	0.033
Dysarthria	−1.135	0.321	0.096–1.078	0.066
Hemiparesis	0.978	2.659	1.325–5.337	0.006
Intercept	−4.243	0.014		< 0.001

*OR* Odds ratio, *CI* Confidence interval

## Discussion

In our study, we found high-grade, ipsilateral arterial pathologies in around 23% of all patients with TIA diagnosed by acute CTA and a need for invasive treatment in around 7% of cases. These numbers indicate that CTA in the acute setting of TIA is justified in selected patient subgroups. As predictors, we identified cardiovascular disease and a short period of symptoms which might be useful for patient selection. In contrast, established TIA-scores turned out to be not predictive for high-grade vascular pathologies.

TIA and ischemic stroke, both indistinguishable in the acute setting in most cases, require vascular imaging. Besides CTA, there are other imaging methods to be considered for neurovascular status evaluation like magnetic resonance imaging and neurovascular ultrasound. Neurovascular ultrasound of the arteries supplying the brain has a high spatial resolution and can detect atherosclerotic changes as well as other vascular pathologies with high accuracy. However, it is limited by investigator dependency and availability. Neurovascular ultrasound in the acute setting can also result in treatment delays due to longer investigation times and patient factors like incompliance, anatomical problems like insufficient transtemporal doppler window and the fact that distal intracranial arteries as well as distal parts of the internal carotid arteries cannot be visualized [17]. Therefore, neurovascular ultrasound is not performed on a regular basis in the acute phase of ischemic stroke if fast and targeted therapy like IVT and EVT is available [18]. Magnetic resonance imaging (MRI) is one of the most important methods to assess the brain tissue. However, it has been shown that CT combined with CTA provides a comparable quality to diffusion-weighted MRI in stroke patients [19]. Considering that MRI resources are more limited compared to CT, which is available 24/7 in all stroke centres, is significantly faster and has a high sensitivity for the diagnosis of vessel occlusions and haemorrhages, CT imaging is the technology of choice in the acute setting of stroke [20]. Considering all advantages and disadvantages of the stroke imaging modalities, it should be noted that vascular imaging should be performed as soon as possible in all suspected TIA and stroke cases.

The evaluated ABCD_2_-, ABCD_3_-score and SPI-II, which all stratify the risk for ischemic stroke after TIA, were not predictive for ipsilateral vascular pathologies or the need for invasive treatment in our study. This observation corresponds to findings from Lou et al. investigating the ABCD_2_-Score as a possible predictor for an interventional treatment. In their study, which included 121 patients with TIA, they found that the ABCD_2_-Score was equally distributed in patients no matter whether they received an intervention or not [21]. Reasons for the missing predictive value of these TIA-scores might be that they have been designed to predict recurrence rates of TIA or ischemic stroke rather than to predict the underlying aetiology, which include various mechanisms. Many factors incorporated in these scores (admission blood pressure, age, diabetes mellitus and heart failure) were equally distributed in our cohort, raising the suspicion that these factors are likely to be usable to quantify instability (risk of recurrence) of the underlying pathology, but are not predictive for the underlying pathology itself. Also, most items are related to the neurologic symptoms presented at admission. As the most important predictors for vascular disease like chronic arterial hypertension, hyperlipidaemia, smoking and other vascular diseases are not considered in these scores, this might explain the lack of their predictive value [22, 23].

Our results suggest that it might be beneficial to take a certain risk profile into account when deciding whether to perform a CTA in the acute setting of TIA or not. Patients with coronary heart disease have a high risk for macroangiopathic vessel disease which again is highly associated with TIA; our data suggests that these patients – especially if combined with a male sex and arterial hypertension - should receive CTA in the acute setting of TIA to diagnose high-risk vascular pathologies and to initiate targeted therapy. CTA in this case could contribute to a gain of time to diagnosis and could contribute to reduce the risk for TIA recurrence or a manifest ischemic stroke. For example, patients with symptomatic high-grade stenosis and poor vascular flow and collateralisation status are at high risk of early disabling stroke [24]. A CTA could reveal the stenosis as well as collateral status and therefore guide targeted blood pressure therapy. In addition, a study by Hause et al. identified de novo dual antiplatelet therapy as a significant predictor for spontaneous recanalization in patients with symptomatic acute extracranial carotid occlusion [25]. Other studies revealed that dual antiplatelet therapy after TIA and minor stroke reduces the risk of recurrent stroke [26–28], which led to the initiation of early dual antiplatelet therapy in current practice. Therefore, the early, targeted initiation of dual antiplatelet therapy can be reassured in patients with an early diagnosis of high-grade, arterial pathologies of the brain supplying vessels in the context of TIA. In this patient group, this early secondary prophylaxis strategy might have the highest impact (e.g. in contrast to in patients with undiagnosed, intermittent atrial fibrillation). However, taking all these findings into account, to date there is no study demonstrating a benefit concerning functional outcome measures in patient with- or without early imaging with CTA. Whether the performance of CTA in the acute setting of TIA translates into outcome improvements must be addressed by future prospective studies.

A short duration of symptoms in TIA-patients is likely to be associated with macroangiopathic arterial disease of brain supplying arteries and can be found in most patients with carotid artery stenosis. A prototype of a short-lasting neurologic deficit associated with carotid artery stenosis is the amaurosis fugax, a special form of TIA [29, 30]. Already in the seventies, Pessin et al. and Harrison et al. found that TIA-patients with symptoms lasting less than 1 h were more likely to have a carotid artery stenosis and should get an angiography [31, 32]. These results are in line with our finding, that a short duration of symptoms increases the odds to diagnose a high-grade, ipsilateral vascular pathology in patients with TIA. In contrast, cardiac emboli are more likely to cause permanent and more severe focal neurological deficits [33–35]. The difference in duration of symptoms caused by arterial or cardiac emboli could be explained by a difference in spontaneous revascularization due to a variation of composition and size of the thrombi [32, 36, 37]. Another point to consider is the collateralisation status in patients with arterial emboli caused by vessel stenosis compared to cardiac embolization with no associated vessel stenosis. Arterial stenosis formation take month to years to develop and induces intracranial collateralization, which is not present in cardiac embolization leading to an acute haemodynamic deficit of brain perfusion. This fact could explain the short symptom duration in patients with arterial stenosis, where cerebral hemodynamics can adapt in a few seconds [38]. Therefore, short duration of symptoms should not be discarded as trivial by the clinician but raise the suspicion of macroangiopathic disease with associated findings in CTA. Concerning the timely development of symptoms, TIA “in crescendo” was previously shown to significantly correlate with large artery atherosclerosis as the origin of TIA [39]. However, this association couldn’t be investigated in our study, as the dynamic and evolution of symptoms were not recorded by the treating physicians.

In addition, our analysis showed that vertigo as presenting symptom of TIA patients predicts relevant vascular pathologies. Although vertigo is a frequent and unspecific symptom in the emergency department, it could be caused by hemodynamic disturbances and impaired cerebral perfusion in patients with stenosis and occlusions of brain supplying arteries. As with a short duration of presenting symptoms, based on our finding the clinician should also pay attention to this unspecific symptom, since it could increase the odds to diagnose relevant vascular findings on CTA in TIA patients.

A strength of our study is the high number of included patients as well as the use of “real word” data from a large volume, tertiary stroke centre. Limitations include the monocentric, retrospective design of the study. Moreover, a selection bias must be considered, as only 66% of patients with TIA received a CTA in the emergency department. The reason for the decision to perform or not to perform a CTA in the acute setting could be influenced by multiple factors like preference of the consultant neurologist in charge, patient factors like kidney disease and medical history of the patient. Another weakness is the lack of MRI data. Therefore, we must assume that our patient population is a mixture of TIA and minor-stroke patients. If more MRI data had been available, the results of our study might be different. However, our data represents everyday clinical praxis and the frequent lack of MRI imaging in even large stroke centres [40].

The results of our study warrant further prospective research on important information like the follow up on patients and how CTA influences their therapy and outcome compared to other imaging methods like neurovascular ultrasound. Further prospective studies could also be used to develop risk scores defining cut-off values to identify patients likely to benefit from acute imaging with CTA.

## Conclusion

In this study, CTA revealed high-grade ipsilateral pathology in more than every fifth patient with TIA and therefore represents an important diagnostic tool in initial workup. History of coronary heart disease, a short duration of symptoms and vertigo as presenting symptom may be useful in triaging TIA patients for acute CTA. Our findings justify performing a prospective, randomized trial that compares patients who receive early neurovascular ultrasound, early CTA and usual care. We want to emphasise that every patient presenting with a persistent acute focal neurologic deficit in emergency department needs immediate vascular imaging, as at this stage a TIA is indistinguishable from a stroke favouring CT-based imaging.

## Supplementary Information

**Additional file 1: Supplementary Table 1.** Presenting symptoms of TIA-patients with- and without ipsilateral vascular pathology and the need for invasive treatment (*n* = 812).

**Additional file 2: Supplementary Table 2.** Univariate and multivariate, binary logistic regression analysis of predictive scores for ipsilateral vascular pathology and the need for invasive treatment in patients with transient ischemic attack.

## Data Availability

All data generated or analysed during this study are included in this published article.

## Abbreviations

CAS: Carotid artery stenting
CEA: Carotid endarterectomy
CTA: Computed tomography angiography
IVT: Intravenous thrombolysis
MRI: Magnetic resonance imaging
MT: Mechanical thrombectomy
PTA: Percutaneous transluminal angioplasty
SPI-II: Stroke prognosis instrument II
TIA: Transient ischaemic attack
