# Supplementary material for: What is the added value of CT-angiography in patients with transient ischemic attack?
Source: BMC Neurol. 2022 Jan 3;22:7. doi: 10.1186/s12883-021-02523-y (PMC8722154; doi:10.1186/s12883-021-02523-y)
Supplement: Supplementary file 1 — Additional file 1: Supplementary Table 1. Presenting symptoms of TIA-patients with- and without ipsilateral vascular pathology and the need for invasive treatment (n = 812). [file 12883_2021_2523_MOESM1_ESM.docx]

| Supplementary table 1: Presenting symptoms of TIA-patients with- and without ipsilateral vascular pathology and the need for invasive treatment (n=812) | | | | | | | |
| --- | --- | --- | --- | --- | --- | --- | --- |
|  |  |  |  |  |  |  |  |
|  | No ipsilateral vascular pathology (n=691) | Ipsilateral vascular pathology (n=121) | p-value |  | No invasive treatment (n=776) | Invasive treatment (n=36) | p-value |
| Hemiparesis (n, %) | 241 (34.9) | 47 (38.8) | 0.400 |  | 269 (34.7) | 19 (52.8) | **0.026** |
| Facial palsy (n, %) | 129 (18.7) | 21 (17.4) | 0.731 |  | 143 (18.4) | 7 (19.4) | 0.878 |
| Dysarthria (n, %) | 155 (22.4) | 26 (21.5) | 0.818 |  | 178 (22.9) | 3 (8.3) | **0.040** |
| Hemihypesthesia (n, %) | 142 (20.5) | 27 (22.3) | 0.766 |  | 160 (20.6) | 9 (25.0) | 0.785 |
| Aphasia (n, %) | 201 (29.1) | 27 (22.3) | 0.126 |  | 217 (28) | 11 (30.6) | 0.735 |
| Double vision (n, %) | 38 (5.5) | 3 (2.5) | 0.162 |  | 41 (5.3) | 0 (0) | 0.157 |
| Hemianopsia (n, %) | 38 (5.5) | 5 (4.1) | 0.536 |  | 41 (5.4) | 2 (5.6) | 0.943 |
| Amaurosis fugax (n, %) | 67 (9.7) | 14 (11.6) | 0.526 |  | 74 (9.5) | 7 (19.4) | **0.052** |
| Vertigo (n, %) | 131 (19.0) | 31 (25.6) | 0.222 |  | 159 (20.5) | 3 (8.3) | 0.197 |
| Cephalgia (n, %) | 48 (6.9) | 9 (7.4) | 0.845 |  | 55 (7.1) | 2 (5.6) | 0.725 |
| Loss of consciousness (n, %) | 20 (2.9) | 6 (5.0) | 0.234 |  | 25 (3.2) | 1 (2.8) | 0.882 |
| TIA: transient ischemic attack | | | | | | | |
|  |  |  |  |  |  |  |  |
