# Supplementary material for: What is the added value of CT-angiography in patients with transient ischemic attack?
Source: BMC Neurol. 2022 Jan 3;22:7. doi: 10.1186/s12883-021-02523-y (PMC8722154; doi:10.1186/s12883-021-02523-y)
Supplement: Supplementary file 2 — Additional file 2: Supplementary Table 2. Univariate and multivariate, binary logistic regression analysis of predictive scores for ipsilateral vascular pathology and the need for invasive treatment in patients with transient ischemic attack. [file 12883_2021_2523_MOESM2_ESM.docx]

| Supplementary table 2: Univariate and multivariate, binary logistic regression analysis of predictive scores for ipsilateral vascular pathology and the need for invasive treatment in patients with transient ischemic attack | | | | | | | | |
| --- | --- | --- | --- | --- | --- | --- | --- | --- |
|  |  |  |  |  |  |  |  |  |
|  |  | Univariate logistic regression | | |  | Multivariate logistic regression | | |
|  |  | OR | 95%CI | p-value |  | OR | 95%CI | p-value |
| Ipsilateral vascular pathology | |  |  |  |  |  |  |  |
|  | ABCD_2_ | 0.930 | 0.820-1.056 | 0.264 |  | 0.901 | 0.791-1.026 | 0.116 |
|  | Sex |  |  |  |  | 1.482 | 0.987-2.227 | 0.058 |
|  | Coronary heart disease |  |  |  |  | 2.059 | 1.282-3.306 | 0.003 |
|  | History of ischemic stroke |  |  |  |  | 1.476 | 0.940-2.320 | 0.091 |
|  | ABCD_3_ | 0.967 | 0.855-1.094 | 0.595 |  | 0.945 | 0.832-1.073 | 0.383 |
|  | Sex |  |  |  |  | 1.495 | 0.995-2.246 | 0.053 |
|  | Coronary heart disease |  |  |  |  | 2.000 | 1.248-3.205 | 0.004 |
|  | History of ischemic stroke |  |  |  |  | 1.441 | 0.919-2.261 | 0.112 |
|  | SPI-II | 1.052 | 0.985-1.123 | 0.135 |  | 1.012 | 0.940-1.091 | 0.743 |
|  | Sex |  |  |  |  | 1.538 | 1.025-2.308 | 0.038 |
|  | Coronary artery disease |  |  |  |  | 1.921 | 1.139-3.238 | 0.014 |
| Need for intervention | | | |  |  |  |  |  |
|  | ABCD_2_ | 0.962 | 0.772-1.198 | 0.727 |  | 1.002 | 0.797-1.260 | 0.987 |
|  | Hyperlipidaemia |  |  |  |  | 5.655 | 0.763-41.905 | 0.090 |
|  | Atrial fibrillation |  |  |  |  | 0.363 | 0.108-1.228 | 0.103 |
|  | TIA within the last seven days |  |  |  |  | 2.136 | 0.949-4.806 | 0.067 |
|  | Antiplatelet medication |  |  |  |  | 1.928 | 0.959-3.876 | 0.066 |
|  | ABCD_3_ | 1.070 | 0.862-1.328 | 0.542 |  | 1.066 | 0.854-1.329 | 0.573 |
|  | Hyperlipidaemia |  |  |  |  | 5.835 | 0.789-43.173 | 0.084 |
|  | Atrial fibrillation |  |  |  |  | 0.335 | 0.100-1.125 | 0.077 |
|  | Antiplatelet medication |  |  |  |  | 1.891 | 0.941-3.798 | 0.074 |
|  | SPI-II | 0.975 | 0.864-1.100 | 0.680 |  | 0.970 | 0.846-1.111 | 0.656 |
|  | Hyperlipidaemia |  |  |  |  | 5.644 | 0.762-41.796 | 0.090 |
|  | Atrial fibrillation |  |  |  |  | 0.389 | 0.113-1.341 | 0.135 |
|  | TIA within the last seven days |  |  |  |  | 2.076 | 0.931-4.629 | 0.074 |
|  | Antiplatelet medication |  |  |  |  | 2.055 | 0.981-4.306 | 0.056 |
| OR: odds ratio, CI: confidence interval, TIA: transient ischemic attack, SPI-II: stroke prognosis instrument; Factors included in the multivariate logistic regression model were confounder predictive with a p≤0.100 in a univariate logistic regression. Factors already included in the prognostic scores, factors causing multicollinearity and factors not known on admission were excluded. | | | | | | | | |
|  |  |  |  |  |  |  |  |  |
